# The prevalence of canine *Leishmania infantum *infection in Sichuan Province, southwestern China detected by real time PCR

**DOI:** 10.1186/1756-3305-4-173

**Published:** 2011-09-12

**Authors:** Li-min Shang, Wei-ping Peng, Hong-tao Jin, Ding Xu, Ni-na Zhong, Wen-long Wang, You-xi Wu, Quan Liu

**Affiliations:** 1 Institute of Military Veterinary, Academy of Military Medical Sciences, Key Laboratory of Jilin Province for Zoonosis Prevention and Control, 666 Liuying Xilu, Changchun 130122, Jilin Province, People's Republic of China; 2 College of Veterinary Medicine, Sichuan Agricultural University, 46 Xingkang Road, Ya'an 625014, Sichuan Province, People's Republic of China; 3 Mianyang Center for Animal Disease Control and Prevention, 62 Fucheng Road, Mianyang 62100, Sichuan Province, People's Republic of China

**Keywords:** *Leishmania*, Dogs, Prevalence, Real time PCR, China

## Abstract

**Background:**

Visceral leishmaniasis (VL) is endemic in western China, and becoming an important public health concern. Infected dogs are the main reservoir for *Leishmania infantum*, and a potential sentinel for human VL in endemic areas. In the present study we investigated the prevalence of *Leishmania *DNA in dogs from Wenchuan, Heishui and Jiuzhaigou County in Sichuan Province, southwestern China, which are important endemic areas of zoonotic VL, detected by real time PCR. The results will help to design control strategies against visceral leishmaniasis in dogs and humans.

**Results:**

The overall prevalence of *Leishmania *DNA in dogs was 24.8% (78/314) in Sichuan Province, with the positive rate of 23.5% (23/98) in Wenchuan County, 28.2% (20/71) in Heishui County, and 24.1% (35/145) in Jiuzhaigou County, and no significant difference was observed among the three counties (*P *> 0.05). The dogs were further allocated to different groups based on sexes, ages and external clinical symptoms. The logistic regression analysis revealed that a higher prevalence was found in older and external symptomatic dogs, compared to that of younger and asymptomatic dogs (*P *< 0.05).

**Conclusions:**

The results revealed that *L. infantum *infection in dogs is widespread in Sichuan Province, southwestern China, which has a public health significance, due to its contribution to the transmission of the infection to humans by sandflies. It is necessary to take measures, including treatment or eradication of infected dogs, to control canine leishmaniasis, which could be helpful to reduce human VL in this area.

## Background

Visceral leishmaniasis (VL) is a severe sandfly-transmitted parasitic disease in humans caused by protozoans of the *Leishmania donovani *complex. The disease is endemic in Europe, Asia, Africa and Latin America, and there are about 500,000 new cases per year in the world [1]. VL has also become an important opportunistic infection related to HIV [2,3]. VL is still an important infectious disease in China, and can be divided into three types: natural focal visceral leishmania (NVL), anthroponotic visceral leishmaniasis (AVL) and zoonotic visceral leishmania (ZVL) [4]. NVL is distributed in Xingjiang and Inner Mongolia, where the wild animals are the sources of infection [5,6]. AVL is transmitted between humans in Shandong, Jiangsu, Hebei, Anhui Province, where patients are the main sources of infection [7]. This type of disease has not been found since 1970s, but there are some sporadic cases of cutaneous leishmaniasis in these regions. ZVL is mainly distributed in Gansu, Qinghai, Ningxia, Sichuan, Shanxi, where canine leishmaniasis caused by *L. infantum *is the important source of human VL [8-10].

Infected dogs are the main reservoir for *Leishmania infantum*, and can be used as a potential sentinel for human VL in the endemic areas [11,12]. Accurate detection of canine leishmaniasis is valuable to prevent transmission to humans. Due to the variable signs of canine leishmaniasis, the clinical diagnosis is not easy [13]. It has been demonstrated that both symptomatic and asymptomatic dogs infected with the parasite are the sources of infection for humans transmitted by the bite of sandfly [10,14]. Therefore, surveillance of canine *L. infantum *infection in endemic areas is very important to control VL in humans and animals.

Serological and PCR-based methods have been widely used to investigate canine infection with *L. infantum*, and PCR-based methods are more sensitive than serological methods [15-19]. Western Sichuan Province, China, including Wenchuan, Heishui and Jiuzhaigou, is an important ZVL endemic area, and also popular with tourists. However, there has been no comprehensive epidemiological survey of canine leishmaniasis in these areas, except a recent study conducted in Jiuzhaigou County [10]. In this study we investigated the prevalence of *Leishmania *DNA in dogs in the three counties by real time PCR.

## Materials and methods

### Study site

The study was carried out in Wenchuan County (30°45'-31°43' N, 102°51'-103°44' E), Heishui County (31°35'-32°38' N, 102°35'-103°30' E) and Jiuzhaigou County (32°53'-33°32' N, 103°27'-104°26' E), located in the mountainous area of western Sichuan Province, southwestern China, where VL is endemic and dogs are important reservoirs for the parasite. Wenchuan County has an annual mean temperature of 13.5~14.1°C, with the mean annual rainfall of 529~1,332 mm and mean altitude of 1,325 meters above sea level. Heishui County has an annual mean temperature of 9.5°C, with the mean annual rainfall of 620 mm and mean altitude of 3,544 meters above sea level. Jiuzhaigou County has an annual mean temperature of 7.3°C, with the mean annual rainfall of 700~800 mm and altitude of 1,140~2,000 meters above sea level. There is a population of 42,600 in Jiuzhaigou, 105,500 in Wenchuan, and 57,000 in Heishui, respectively. In recent years, there were about 20, 10 and several human VL cases reported in Jiuzhaigou, Wenchuan, and Heishui per year, respectively [20]. *Phlebotomus chinensis *is the predominant species as well as the important transmission vector of VL in western Sichuan Province, southwestern China, and the average prevalence of *Leishmania *parasites in *P. chinensis *females is 1.98% [21].

### Animals and sampling

The protocol for sampling was reviewed and approved by the Animal Ethics Committee of Institute of Military Veterinary, Academy of Military Medical Sciences (MVI2009-107). Oral consent was obtained from the owners of dogs. There were 5,813, 4,938 and 8,875 dogs in Wenchuan, Heishui and Jiuzhaigou County, respectively. The household dogs were randomly collected at the capital town of the county and adjacent villages in May and June, 2010, and examined for the external clinical signs of the diseases, including weight loss, dry exfoliative dermatitis, ulcers, periorbital alopecia, diffuse alopecia and ocular signs. Two milliliters of blood samples were taken from the foreleg vein of each dog in EDTA-coated polypropylene tubes for isolation of parasite DNA.

### DNA extraction

The *Leishmania *isolate MHOM/CN/92/SC10H2 was originally obtained from Sichuan Province and maintained in our laboratory [22]. The parasite was grown in NNN medium at 24°C for 14 days. DNA extraction from cultured parasite and dog blood samples was carried out using TIANamp Genomic DNA Kit for blood, cell and tissue (Tiangen Biotech Co., LTD, Beijing, China) according to manufacturer's instruction. The extracted DNA was suspended in elution buffer (10 mM Tris, 1 mM EDTA, pH8.0).

### Real time PCR

*Leishmania *DNA in peripheral blood was detected by real time PCR using SYBR^® ^Green Real-time PCR Master Mix-Plus (Toyobo Biotechnology) as previously described [23]. The real time PCR was carried out using the primers (forward: 5'-CCTATTTTACACCAACCCCCAGT-3'; reverse: 5'-GGGTAGGGGCGTTCTGCGAAA-3') that amplify the 120 bp fragment of the minicircle kinetoplast DNA of *Leishmania*. The limit of detection was 0.1 parasite per real time PCR reaction. Genomic DNA from *Leishmania *reference strain MHOM/CN/92/SC10H2 was used as a positive control, and the negative control was established with deionized water instead of DNA extract.

### Statistical analysis

Differences in prevalence of dogs from different sampling sites and among associated factors were analyzed using the Chi square test and logistic regression by SPSS version 11.0 software. The difference was considered statistically significant when *P *< 0.05.

## Results

### *Prevalence of Leishmania *DNA in dogs

A total of 314 household dogs were chosen for real time PCR detection of *Leishmania *DNA, and 78 (24.8%) of 214 were found *Leishmani*a-positive in Sichuan Province, southwestern China, with 23 (23.5%) of 98 in Wenchuan, 20 (28.2%) of 71 in Heishui, and 35 (24.1%) of 145 in Jiuzhaigou tested positive (Table 1). No significant difference was observed in prevalence among the three counties (*P *> 0.05).

**Table 1:** Prevalence of *Leishmania *infection in dogs from the three counties of Sichuan Province, China

Region	No. of examined	No. of positive	Prevalence (%)
Wenchuan	98	23	23.5
Heishui	71	20	28.2
Jiuzhaigou	145	35	24.1
Total	314	78	24.8

### Risk factors for *Leishmania *infection in dogs

The dogs were further allocated to different groups, based on sexes, ages and clinical symptoms of VL. These groups included male (n = 176) or female (n = 138), the ≤1 year old (n = 220) or the > 1 year old (n = 94); and the asymptomatic (presenting no external clinical signs of VL; n = 241) or the symptomatic (presenting one of the external clinical signs of VL, such as weight loss, dry exfoliative dermatitis, ulcers, periorbital alopecia, diffuse alopecia and ocular signs; n = 73) (Table 2).

**Table 2:** Risk factors for *Leishmania *infection in dogs from Sichuan Province, China

Data of dogs	No. of examined	No. of positive	Prevalence (%)	*P*	OR (95.0% CI)
Sex					
Male	176	44	25.0	0.941	0.5 (0.2-1.0)
Female	138	34	24.6		
Age					
≤ 1 year	220	43	19.5	0.001	2.4 (1.4-4.2)
> 1 year	94	35	37.2		
Clinical status ^a^					
Symtomatic	73	26	35.6	0.015	2.0 (1.1-3.6)
Asymtomatic	241	52	21.6		

^a ^The clinical status including the asymptomatic dogs, which present no clinical signs of VL, and symptomatic dogs, which present one of the external clinical signs of VL, such as weight loss, dry exfoliative dermatitis, ulcers, periorbital alopecia, diffuse alopecia and ocular signs.

There was no any significant association between sex and the prevalence of infection (*P *= 0.941). However, a significant association between dog age and prevalence of VL was found (*P *= 0.001), with a higher prevalence in > 1 year old group (37.2%) than that in ≤ 1 year old group (19.5%). Dogs aged > 1 year old had a much higher risk of acquiring VL infection than those aged ≤ 1 year old (OR = 2.4; 1.4-4.2 CI 95%). The prevalence (35.6%) of dogs with external clinical signs was higher when compared with that (21.6%) of dogs in which external clinical signs were not found (Table 2).

## Discussion

ZVL caused by the kinetoplastid protozoan *L. infantum *is still endemic in China, especially in the northwest regions [24,25]. The parasite is transmitted to humans by the bite of the sandfly *Phlebotomus chinensi*, and infected dogs serve as the main reservoir [9,10]. Standard measures to control ZVL in China have depended on eradication of infected dogs, vector control and treatment of patients, and these measures have played important roles to prevent the rapid rise and spread of ZVL in China [26]. However, the increased prevalence of *L. infantum *in dogs would cause local outbreak of ZVL in these regions, and eradication of dogs can markedly reduce the number of human cases [27]. Therefore, the prevalence of *L. infantum *infection in dogs would show trends of ZVL in humans [11,28,29]. In this study we collected blood samples from dogs in endemic areas of Wenchuan, Heishui and Jiuzhaigou County, China and evaluated the prevalence of *L. infantum *infection in these dogs by real time PCR

Based on our results, 24.8% dogs were detected positive for *Leishmania*, with the prevalence of 23.5% in Wenchuan, 28.2% in Heishui and 24.1% in Jiuzhaigou, less than those reported by other studies in high endemic areas [10,30-32], while higher than those studies conducted in low endemic regions [33-35]. The results are related to the samples of different endemic areas.

We found that age and external clinical status of dogs are associated with prevalence of *Leishmania *infection. Dogs aged > 1 year old had higher prevalence of *L. infantum *infection than those aged ≤ 1 year old, resulting from most likely longer exposure to infective sandfly bites. The dogs with external clinical signs, such as weight loss, dry exfoliative dermatitis, ulcers, periorbital alopecia, diffuse alopecia and ocular signs also had higher prevalence than the asymptomatic dogs. The possible reason would be that the symptomatic dogs have higher parasite load, resulting in a higher detection rate [36]. Our results showed that dogs with no external clinical signs may also harbor *Leishmania*, although in a lower prevalence as compared to dogs with external clinical signs [37,38].

The serological and PCR-based methods are sensitive and specific to investigate canine leishmaniasis. For detection in symptomatic dogs, the sensitivity of two methods is comparable. But for asymptomatic dogs, the PCR method is more sensitive than serological methods [39-41]. The probable reason is that asymptomatic infection dogs have low parasite burden and antibody levels, while the detection limit of PCR can be a single parasite. Our results, combined with other studies, demonstrated that real time PCR is a reliable method to detect *Leishmania *DNA in dogs. However, a comprehensive evaluation of PCR combined with clinicopathological and serological results is important to diagnose canine leishmaniasis [42].

## Conclusions

The results of the present survey revealed that *L. infantum *infection in dogs is widespread in Sichuan Province, southwestern China, which has public health significance due to its contribution to the transmission of the infection to humans by sandflies. It is necessary to implement strategies, including treatment or eradication of infected dogs, to control canine leishmaniasis, which could help to reduce human VL in this area.

## Competing interests

The authors declare that they have no competing interests.

## Authors' contributions

LS and WP carried out real time PCR. WP, DX, NZ, WW and YW collected the samples. HJ and NZ performed data analysis. QL and NZ designed the study. QL drafted the manuscript. All authors read and approved the final manuscript.
